# Hepatic Metabolic Derangements Triggered by Hyperthermia: An In Vitro Metabolomic Study

**DOI:** 10.3390/metabo9100228

**Published:** 2019-10-15

**Authors:** Ana Margarida Araújo, Maria Enea, Félix Carvalho, Maria de Lourdes Bastos, Márcia Carvalho, Paula Guedes de Pinho

**Affiliations:** 1 UCIBIO, REQUIMTE, Laboratory of Toxicology, Faculty of Pharmacy, University of Porto, Rua Jorge Viterbo Ferreira, 228, 4050-313 Porto, Portugal; eneavmaria@gmail.com (M.E.); felixdc@ff.up.pt (F.C.); mlbastos@ff.up.pt (M.d.L.B.); 2 UFP Energy, Environment and Health Research Unit (FP-ENAS), University Fernando Pessoa, Praça Nove de Abril, 349, 4249-004 Porto, Portugal

**Keywords:** heat stress, primary mouse hepatocytes, metabolic profile, GC-MS, multivariate statistical analysis

## Abstract

Background and aims: Liver toxicity is a well-documented and potentially fatal adverse complication of hyperthermia. However, the impact of hyperthermia on the hepatic metabolome has hitherto not been investigated. Methods: In this study, gas chromatography-mass spectrometry (GC-MS)-based metabolomics was applied to assess the in vitro metabolic response of primary mouse hepatocytes (PMH, *n* = 10) to a heat stress stimulus, i.e., after 24 h exposure to 40.5 °C. Metabolomic profiling of both intracellular metabolites and volatile metabolites in the extracellular medium of PMH was performed. Results: Multivariate analysis showed alterations in levels of 22 intra- and 59 extracellular metabolites, unveiling the capability of the metabolic pattern to discriminate cells exposed to heat stress from cells incubated at normothermic conditions (37 °C). Hyperthermia caused a considerable loss of cell viability that was accompanied by significant alterations in the tricarboxylic acid cycle, amino acids metabolism, urea cycle, glutamate metabolism, pentose phosphate pathway, and in the volatile signature associated with the lipid peroxidation process. Conclusion: These results provide novel insights into the mechanisms underlying hyperthermia-induced hepatocellular damage.

## 1. Introduction

Thermoregulation is a complex process, crucial for body homeostasis and survival, that is meticulously orchestrated by the thermoregulatory center in the hypothalamus [1]. A failure in hypothalamic regulation leads to an imbalance between heat accumulation (either due to extreme environmental temperatures and/or body heat generation) and heat dissipation, and may cause a huge increase in body temperature above that considered physiologically normal—this condition is commonly referred as hyperthermia [2]. A wide variety of xenobiotics can affect the thermal homeostasis, triggering or exacerbating the hyperthermia-induced damage, both by the increased metabolic heat production (e.g., sympathomimetic agents) or by an impairment of heat-dissipating effector mechanisms (e.g., anticholinergic agents) [3]. This disruption will consequently affect many other homeostatic systems and may result in several life-threatening complications such as disseminated intravascular coagulation, hyperkalemia, metabolic acidosis, multi-organ failure, and rhabdomyolysis [4,5,6].

Hepatocellular injury is a well-documented adverse complication of heat stroke, and oxidative stress has been identified as the main mechanism underlying hyperthermia-induced liver toxicity [7,8,9]. Earlier in vitro studies have provided convincing evidence that hyperthermia per se stimulates an aggressive pro-oxidant state in freshly isolated rat and mouse hepatocytes and in rat liver, making liver cells more vulnerable to prooxidant species that subsequently lead to lipid peroxidation and cellular damage [9,10,11,12]. Furthermore, it is also known that variations in the cellular temperature affect the effectiveness of various enzymes and alter membrane stability and diffusion capacity, which disrupts a great number of critical cellular functions, such as energy use and membrane ion fluxes [13]. However, specific hepatic metabolic pathways altered by hyperthermia remain largely unknown.

The aim of this study was to improve our understanding of how hyperthermia affects the cellular metabolome of primary mouse hepatocytes (PMH). For this, a gas chromatography-mass spectrometry (GC-MS) untargeted metabolomic approach was used to analyze the metabolic profile of primary mouse hepatocytes at hyperthermic (40.5 °C) conditions and compare it with normothermic (37 °C) conditions. The analysis of the metabolites released from (extracellular metabolome) or existing within the cells (intracellular metabolome) was performed in order to obtain a more detailed metabolic characterization profile. As far as we know, this is the first metabolomic study to investigate the metabolic derangements triggered by hyperthermia in hepatic cells.

## 2. Results

### 2.1. Hyperthermic Conditions Affect the Viability of PMH

**Figure 1:**
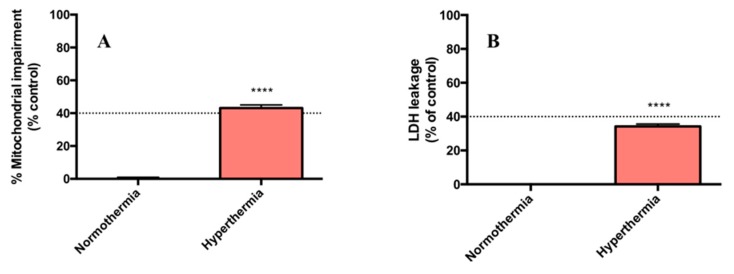
Cell viability measured by (**A**) MTT reduction and (**B**) lactate dehydrogenase (LDH) leakage, 24 h after exposure of primary mouse hepatocytes to normothermic (37 °C) and hyperthermic (40.5 °C) conditions. Results were obtained from 10 independent experiments, performed in triplicate. **** *p* < 1.00 × 10^−4^ (hyperthermic vs. normothermic conditions).

Changes in viability of PMH triggered by hyperthermic conditions were evaluated using the MTT reduction and lactate dehydrogenase (LDH) leakage assays. The data presented in Figure 1A show that a temperature rise from 37 to 40.5 °C, after 24 h, caused a significant reduction in cell viability as compared with control (about 40% decrease in viability according to the MTT reduction assay, *p* < 0.0001) and also significantly affected the cellular membrane integrity, according to the LDH release assay (about 35% of the cells were affected, *p* < 0.0001) (Figure 1B). In order to ensure that pH of the culture medium was not affected by temperature, and therefore contributed to the observed cell death, this factor was measured, and no significant differences were found between groups.

### 2.2. Hyperthermia Significantly Alters the Metabolic Profile of PMH

**Figure 2:**
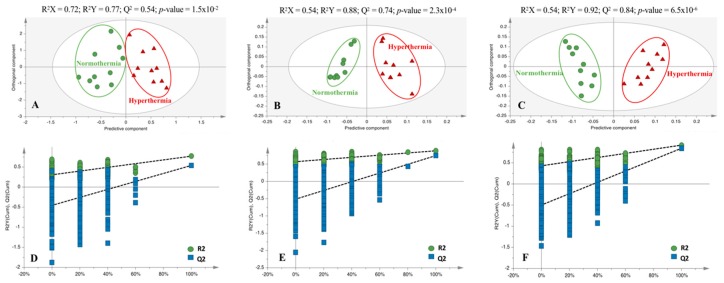
Orthogonal projections to latent structures discriminant analysis (OPLS-DA) score scatter plots obtained for the chromatograms corresponding to cells exposed to normothermic (*n* = 10, ●) and hyperthermic (*n* = 10, ▲) conditions, after analysis of the (**A**) intracellular metabolome, (**B**) volatile organic compound (VOC) and (**C**) volatile carbonyl compound (VCC) in extracellular metabolome. (**D**–**F**) Statistical validation of the respective OPLS-DA models obtained by permutation tests (500 permutations).

Data obtained in this study showed that all quality control (QC) samples and the internal standards used in the analysis of the intracellular and extracellular (volatile organic compounds, VOCs and volatile carbonyl compounds, VCCs) metabolome had good reproducibility over the acquisition time (Figure S1), the chromatographic datasets being considered robust and qualified for statistical analyses. Unsupervised multivariate analysis revealed that the exposure of PMH to heat stress resulted in significant alterations in the intra- and extra-cellular metabolome, since the separation between cells under normothermic and hyperthermic conditions was already apparent in all principal component analysis (PCA) score plots (Figure S2), indicating a unique metabolite profile of each group. This separation was maximized in the orthogonal projections to latent structures discriminant analysis (OPLS-DA) models (Figure 2A–C) which presented good quality parameters (R^2^X = 0.72, R^2^Y = 0.77, Q^2^ = 0.54, and *p*-value = 1.5 × 10^−2^ for intracellular data; R^2^X = 0.54, R^2^Y = 0.88, Q^2^ = 0.74, and *p*-value = 2.3 × 10^−4^ for VOCs, and R^2^X = 0.54, R^2^Y = 0.92, Q^2^ = 0.84, and *p*-value = 6.5 × 10^−6^ for VCCs). Furthermore, the robustness of all OPLS-DA models was confirmed through a permutation test (Figure 2D–F), since all R^2^ and Q^2^ values of the permuted classes are lower than the original classes.

**Figure 3:**
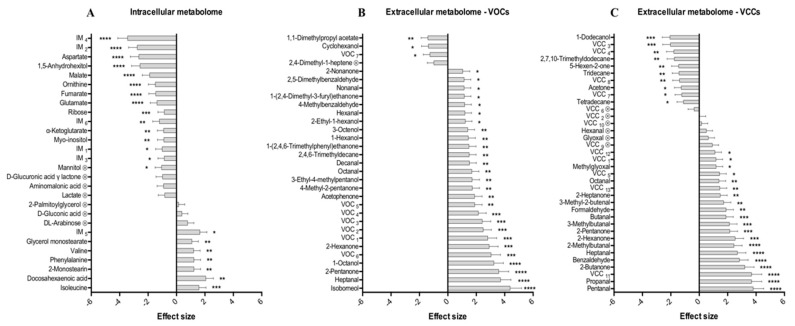
Effect size of the metabolites altered by heat stress, evaluated by comparison of cells exposed to hyperthermic vs. normothermic conditions in the (**A**) intracellular metabolome and (**B** and **C**) extracellular metabolome (VOCs and VCCs, respectively). Unidentified compounds are reported as ‘IM_i_’, ‘VOC_i_’ and ‘VCC_i_’ (i = 1, 2, 3...) according to the ascending order of their retention time (RT) values. Metabolites marked with ⊗ are not statistically significant after false discovery rate (FDR) correction (FDR corrected *p*-value: 3.93 × 10^−2^ for intracellular metabolome, 4.84 × 10^−2^ for VOCs, and 4.12 × 10^−2^ for VCCs). * *p* < 5.00 × 10^−2^, ** *p* < 1.00 × 10^−2^, *** *p* < 1.00 × 10^−3^, **** *p* < 1.00 × 10^−4^ (hyperthermic vs. normothermic conditions).

Through the analysis of the corresponding loading *S*-plot, the variables with VIP > 1 combined with |p(corr)| >0.5 were selected for integration in order to assess the magnitude and significance of metabolic variations caused by hyperthermia (Figure 3). A total of 28 intracellular metabolites were identified as potentially discriminant (|p(corr)| >0.5 and VIP > 1), including several amino acids and derivatives, organic acids, carbohydrates, and fatty acids derivatives. In brief, only 22 intracellular metabolites appeared significantly (*p* < 0.05) affected by the temperature increase, of which 15 were significantly decreased (namely 1,5-anhydrohexitol, fumarate, malate, 2-ketoglutarate, aspartate, glutamate, ornithine, mannitol, myo-inositol, ribose, and five unidentified metabolites) and seven significantly increased (including valine, phenylalanine, isoleucine, docosahexaenoic acid, 2-monostearin, glycerol monostearate, and one unidentified metabolite) (Figure 3A). In parallel, the univariate analysis revealed that of a total of 31 potentially discriminating VOCs (|p(corr)| >0.5 and VIP > 1), 30 varied significantly (*p* < 0.05) between the two groups (hyperthermia vs. normothermia). Significantly altered VOCs include one alkane, one ester, two aldehydes, five alkanals, seven ketones, seven alcohols, and seven unidentified metabolites. In general, VOCs appeared to be significantly up-regulated, with exception of 1,1-dimethylpropyl acetate, cyclohexanol, and an unknown compound which appeared to be significantly down-regulated after temperature rise (Figure 3B). Regarding VCCs analysis, the metabolites found significantly altered in the hyperthermic conditions compared to the normothermic condition are summarized in Figure 3C.

The corresponding loading *S*-plot indicates 36 VCCs as potentially discriminant (|p(corr)| >0.5 and VIP > 1), of which 30 were found significantly altered (*p* < 0.05). Significant metabolites detected after VCCs analysis include an alcohol, an alkenal, a dicarbonyl, an aromatic aldehyde, three alkanes, six ketones, eight alkanals, and nine unidentified metabolites. Univariate analysis indicated that tetradecane, tridecane, 2,7,10-trimethyldodecane, acetone, 1-dodecanol, and four unidentified compounds appeared to be significantly down-regulated in the medium after temperature rise, while all other VCCs appeared to be significantly up-regulated in hyperthermic conditions. It is noteworthy that in the analysis of VOCs and VCCs five common compounds were found (hexanal, heptanal, octanal, 2-pentanone, and 2-hexanone) with the same alteration trends (up-regulation under hyperthermia).

### 2.3. Discriminant Metabolites Identified

A total of 28 intracellular metabolites and 67 extracellular metabolites (31 VOCs and 36 VCCs) were indicated as potentially altered under hyperthermic conditions. A complete list with the information used for the identification of these discriminant metabolites (such as retention time (RT), characteristic ions (*m*/*z*), retention indexes (RI), reverse match score, Human Metabolome Database (HMDB) and Kyoto Encyclopedia of Genes and Genomes (KEGG) identification codes) as well as their identification level is summarized in Tables S1 and S2 [14]. The identification of 44 metabolites was unequivocally confirmed with analytical standards (level 1), 25 metabolites were putatively identified based on commercial spectral libraries (level 2), a compound class was attributed to four metabolites (level 3), and 22 metabolites were not yet identified (level 4) (Table S1 and S2).

### 2.4. Hepatic Biochemical Pathways Affected by Hyperthermia

**Figure 4:**
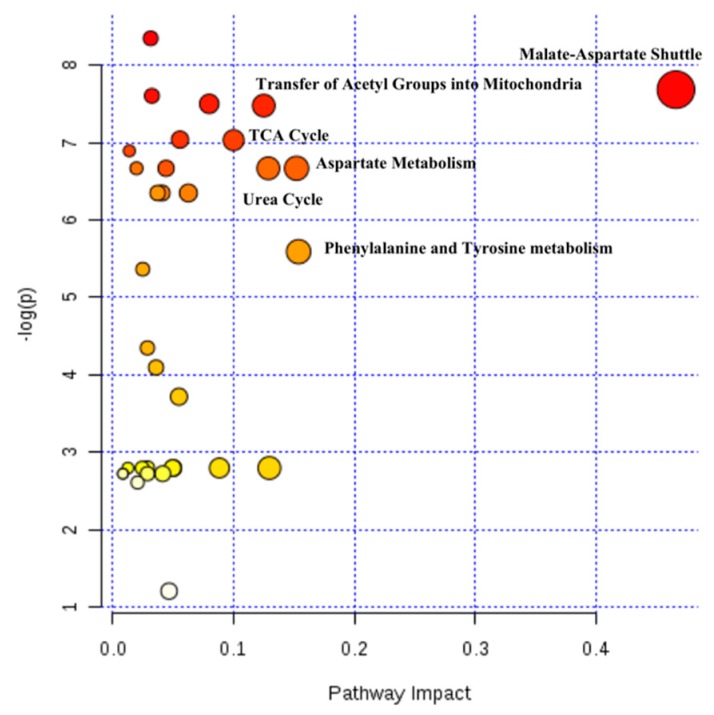
Overview of the dysregulated metabolic pathways based on metabolites alteration caused by hyperthermia. The node color is based on the *p* value, where a dark circle color indicates a more significant pathway. The node radius corresponds to the pathway impact value. Pathways were annotated when *p* < 0.05 and pathway impact > 0.1.

To aid in the identification of the major disturbed metabolic pathways, metabolites significantly altered by temperature (*p* < 0.05) were analyzed using the MetaboAnalyst 4.0 software. Our analysis revealed that phenylalanine and tyrosine metabolism, aspartate metabolism, urea cycle, tricarboxylic acid (TCA) cycle, the transference of acetyl groups into the mitochondria and malate-aspartate shuttle were the hepatic pathways more affected by hyperthermia (Figure 4).

**Figure 5:**
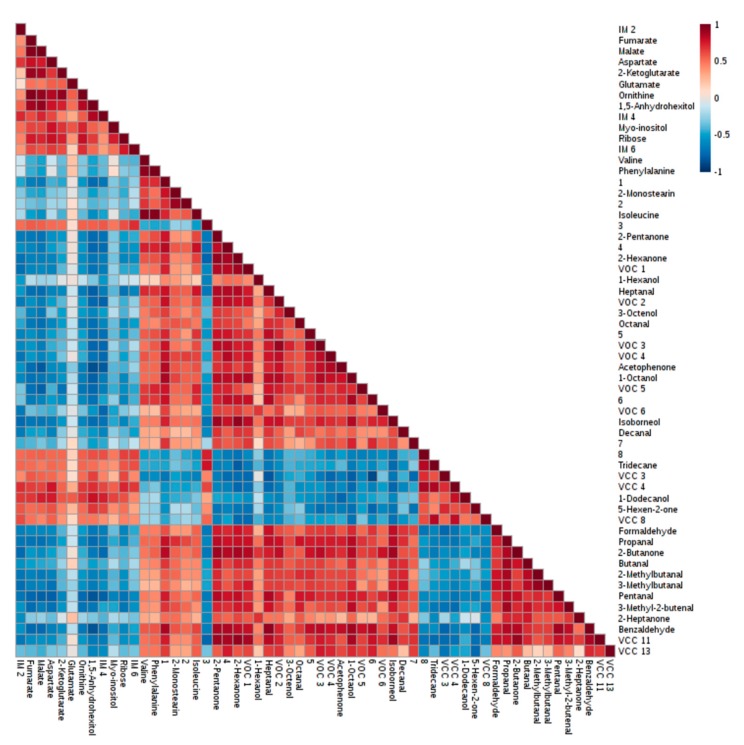
Heatmap representing the Spearman’s correlations between the metabolites significantly altered (*p* < 0.01) after a thermal insult. 1. Docosahexaenoic acid; 2. Glycerol monostearate; 3. 1,1-Dimethylpropyl acetate; 4. 4-Methyl-2-pentanone; 5. 3-Ethyl-4-methylpentanol; 6. 2,4,6-Trimethyldecane; 7. 1-(2,4,6-Trimethylphenyl)ethanone; 8. 2,7,10-Trimethyldodecane.

Due to the limitations associated with the software database and to overcome the lack of knowledge about the role of some compounds in the metabolic pathways (especially volatile compounds), Spearman’s correlation indexes were calculated using all discriminant metabolites (*p* < 0.01) (Figure 5). Taking into account a |correlation index| ≥0.90 and *p* < 0.0001, data revealed the existence of several strong positive correlations, namely between 2-pentanone and VCC_11_ (r = 0.90), 2-pentanone and 2-butanone (r = 0.90), 2-hexanone and VOC_1_ (r = 0.90), fumarate and 2-ketoglutarate (r = 0.91), glycerol monostearate and 2-monostearin (r = 0.91), 2-butanone and VCC_11_(r = 0.91), 2-pentanone and isoborneol (r = 0.91), VOC_2_ and VOC_3_ (r = 0.91), malate and ornithine (r = 0.92), 2-pentanone and 2-hexanone (r = 0.92), malate and 1,5-anhydrohexitol (r = 0.93), 2-hexanone and VCC_11_ (r = 0.93), 2-methylbutanal and 3-methylbutanal (r = 0.93), heptanal and benzaldehyde (r = 0.93), 2-ketoglutarate and ornithine (r = 0.94), valine and phenylalanine (r = 0.94), 2-hexanone and isoborneol (r = 0.94), fumarate and malate (r = 0.96), phenylalanine and isoleucine (r = 0.96), fumarate and ornithine (r = 0.97), and finally between valine and isoleucine (r = 0.97). These correlation magnitudes suggest that these metabolites may share the same metabolic pathway or some common regulatory mechanism. Moreover, the Spearman’s rank correlation coefficient suggests that VCC_11_ may also be a ketone derivative due to their positive correlations with 2-butanone, 2-pentanone and 2-hexanone as well as common characteristic MS fragments (Table S2).

## 3. Discussion

The number of metabolomic studies based on mass spectrometry methodologies has increased exponentially due to its high sensitivity, selectivity, and rapid data acquisition [15]. In this work, different GC-MS approaches were implemented to study the intracellular and extracellular metabolome in order to better characterize the changes caused by hyperthermia in mice isolated hepatocytes. Hyperthermia induced a significant loss of cell viability under our experimental conditions (approximately 35%, according to the LDH assay). This may represent a limitation of the present study as the metabolome reflects both viable and dead cells, but may also be more representative of the real consequences derived from a hyperthermic state, as the resulting liver diseases are also associated with the death of hepatic cells. The combination of intracellular and extracellular datasets clearly demonstrated that an increase in temperature from physiological (37 °C) to hyperthermic (40.5 °C) conditions, after 24 h, induces profound changes in the hepatic metabolome, reflected in the level of 22 intracellular metabolites and 59 extracellular volatile metabolites (30 VOCs and 29 VCCs). This high number of metabolites suggests that several metabolic pathways may be altered and contribute to the liver damage. Therefore, the main hepatic changes described in this study as being induced by hyperthermia will be discussed below.

First of all, our metabolomic study revealed that one of the major alterations caused by hyperthermia was the significant decrease of some TCA cycle intermediates, namely 2-ketoglutarate (*p* < 0.001), fumarate and malate (*p* < 0.0001), suggesting a mitochondrial dysfunction and consequently an energetic failure. Heat stress has been shown to cause mitochondrial protein denaturation, specifically the pyruvate decarboxylase complex subunits and the TCA cycle enzymes, which could explain these decreased levels [16]. Additionally, cells exposed to a thermal insult showed alterations in the levels of some glucogenic and/or ketogenic amino acids, with glutamate and aspartate having a significant depletion (*p* < 0.0001) and phenylalanine (*p* = 0.0034), valine (*p* = 0.0040), and isoleucine (*p* = 0.0005) a significant increase. Protein denaturation may also justify the accumulation of phenylalanine, valine, and isoleucine, since they are not being used to restore the basal levels of TCA cycle intermediates, possibly due to an ineffective activity of transaminases. This enzymatic alteration may contribute to the impairment of the energetic pathway, and although a positive correlation between phenylalanine, valine, and isoleucine support the existence of a common regulatory pathway, further studies are needed to confirm this theory. Moreover, despite the impossibility of contributing to the restoration of the TCA cycle intermediates via transamination, glutamate and aspartate seem to be used in other metabolic pathways, since their intracellular levels are reduced.

As suggested by the pathway analysis, the urea cycle appears to be also affected, since a significant decrease (*p* < 0.0001) in ornithine levels was observed after a rise in the incubation temperature from 37 to 40.5 °C. However, it is possible that the ornithine levels may have been grossly estimated, since arginine can be converted into ornithine during the derivatization reaction and, therefore, some caution in the interpretation of this result is necessary [17].

Another remarkable change observed in this study was the significant decrease of glutamate levels (*p* < 0.0001) in hyperthermic conditions. This metabolite is crucial for the glutathione (GSH) synthesis, since its first biosynthesis phase requires an ATP enzymatic step that leads to the formation of γ-glutamylcysteine from glutamate and cysteine [18]. Thus, low levels of glutamate coupled with the breakdown in ATP production indicate that the ability of hepatocytes to synthesize GSH may be compromised [18]. Glutathione plays several vital functions, including detoxification of xenobiotics and/or their metabolites and cell signaling, and is also a major cellular antioxidant crucial in protecting cells against oxidative stress [18]. Since hyperthermia is a pro-oxidant aggressive condition, decreased levels of hepatic GSH may compromise cellular antioxidant defenses and render cells more susceptible to the deleterious effects of reactive oxygen and nitrogen species (ROS/RNS) formed within the cell [9]. Consistent with our results, a significant reduction of GSH was observed in freshly isolated rat hepatocytes and in the HepG2 cell line after exposure to heat stress [10,19]. Furthermore, it has been demonstrated that GSH depletion triggered by hyperthermic conditions is in fact coupled with an increased ROS/RNS production [19]. Owing to their high reactivity, ROS/RNS can interact with the lipids present in the cell and cause their oxidative damage with consequent lipid peroxidation and impairment of cell membrane functions [20]. Lipid peroxidation gives rise to diverse secondary end-products capable of reacting with several intracellular targets and exert adverse biological effects [20]. The study of the volatile fraction of the extracellular metabolic profile developed in this work detected several of these secondary end-products, where hydrocarbons, alcohols, ketones, and aldehydes appeared as the main classes significantly altered under hyperthermic conditions. Hydrocarbons are a class of compounds typically generated by polyunsaturated fatty acid (PUFA) peroxidation [21,22]. However, in our study their levels were significantly decreased after a temperature increase. Such decrease may be explained by a possible up-regulation of cytochrome P450 enzymes responsible for hydroxylating alkanes and lead to the production of the corresponding alcohols, which, with the exception of cyclohexanol and 1-dodecanol, appear increased under hyperthermic conditions [21,22]. On the other hand, alcohols can be converted into ketones or aldehydes, which could justify the reduced levels of cyclohexanol and 1-dodecanol and the significant increase of some of the ketone and aldehyde metabolites found in the extracellular environment of cells exposed to hyperthermia [21]. The increase in aldehydes may also have a direct origin in the reduction of hydroperoxides by cytochrome P450 [21,22]. Most of the volatile compounds detected in our study are positively correlated, which may suggest a common metabolic origin. These results suggest that lipid peroxidation is responsible for most of the metabolic changes that hyperthermia causes in the volatile fraction of surrounding medium of PMH. In fact, this harmful process had already been associated with the hepatotoxicity caused by heat stress [9].

Finally, another volatile compound found significantly increased after exposure to hyperthermia was formaldehyde (*p* = 0.0230). Formaldehyde may be endogenously formed through the L-methionine, histamine, methanol, and methylamine metabolic pathway, and its catabolism involves its conversion to CO_2_ through reactions involving glutathione [23,24]. However, since increasing temperature seems to compromise the glutathione metabolism, this could explain the endogenous accumulation of formaldehyde, that can rapidly react with nucleophilic groups present in nucleic acids and proteins, leading to mutagenesis and cell death [23]. Low glutathione levels may also represent a limiting factor for methylglyoxal detoxification, which was another volatile compound found with significantly increased levels in the extracellular environment of cells exposed to hyperthermia (*p* = 0.0212) [25]. Although the main production pathway of methylglyoxal is associated with glycolysis, in pathological conditions the oxidation of ketone bodies is also an important source of this compound [26]. In fact, this study showed that this methylglyoxal formation route seems to be activated, since hyperthermia led to a significant decrease (*p* = 0.0129) in acetone levels compared to the basal levels found in normothermia. Methylglyoxal is one of the most potent and reactive glycating agents present in cells, so its accumulation causes several deleterious effects, including genotoxic effects [25]. Evidence suggests that high levels of methylglyoxal causes interstrand cross-links in duplex DNA, strand breaks, and increased mutation frequency [26]. Thus, taking into account that hyperthermia considerably affects DNA repair mechanisms, PMH will be more sensitive to methylglyoxal’s genotoxic effects [16].

In addition, our metabolomic results also suggest a dysfunction of the pentose phosphate pathway since a significant decrease of the ribose reserves was observed (*p* = 0.0003), an intermediate metabolite essential for the nucleotide biosynthesis and that can consequently affect DNA repair mechanisms [27].

In several in vivo studies where the global heat stress was evaluated in different matrices (serum, plasma, urine, milk, liver), alterations in the amino acid metabolism, TCA cycle, and nucleotide metabolism were also found [28,29,30]. Our findings are in agreement with the changes found in in vivo studies, thereby suggesting translatability of our results.

## 4. Materials and Methods

### 4.1. Chemicals

All reagents were of analytical grade or of the highest grade available. Antibiotic mixture of penicillin/streptomycin (10,000 U/mL/10,000 mg/mL), fungizone (250 mg/mL), and heat-inactivated fetal bovine serum (FBS) were obtained from GIBCO Invitrogen (Barcelona, Spain). Collagen G was obtained from Merck (Darmstadt, Germany). 4-Fluorobenzaldehyde (≥98%), collagenase from *Clostridium histolyticum* Type IA, desmosterol (≥84%), dexamethasone, ethylene glycol-bis-(2-aminoethylether)-*N*, *N*, *N*’, *N*’-tetraacetic acid (EGTA), gentamicin, insulin solution from bovine pancreas (10 mg/mL), methoxyamine hydrochloride (≥98%), *N*,*O*-bis(trimethylsilyl)trifluoroacetamide with 1% trimethylchlorosilane (BSTFA + 1% TMCS), *O*-(2,3,4,5,6-pentafluorobenzyl)hydroxylamine hydrochloride (PFBHA, ≥99%), sodium chloride (NaCl, ≥99.5%), thiazolyl blue tetrazolium bromide (MTT, ≥98%), thymol (≥98.5%), Triton X-100, trypan blue solution, Williams’ E medium, and all standards used throughout the work were purchased from Sigma-Aldrich (St. Louis, Missouri, USA). Methanol (≥99.9%) and pyridine (≥99%) were purchased from VWR (Leuven, Belgium).

### 4.2. Isolation and Primary Culture of Mouse Hepatocytes

Ten male CD-1 mice (7–9 weeks old) were used in these experiments. Animal housing and experimental procedures were performed in accordance with Portuguese legislation (Decree-Law No. 113/2013, of August 7th), and approved by the Ethical Committee of the Faculty of Pharmacy of University of Porto (protocol number P158/2016) and by the Portuguese National Authority for Animal Health (reference number 0421/000/000/2017). Isolation of hepatocytes was performed using a modified collagenase perfusion method, as described by Godoy et al. [31]. Surgical procedures were performed under isoflurane anesthesia and carried out between 10.00 and 11.00 a.m. The initial viability of the isolated mouse hepatocytes was estimated by the trypan blue exclusion test and was always greater than 80%. Subsequently, a suspension containing 0.5 × 10^6^ viable cells/mL was prepared in complete culture medium (William’s E medium supplemented with 10% FBS, 100 U/mL penicillin, 100 mg/mL streptomycin, 100 µg/mL gentamicin, 5 µg/mL insulin, 50 nM dexamethasone, and 2.5 µg/mL fungizone) and seeded into a collagen-coated 35-mm Petri dishes (for metabolomic studies) and 96-well culture plates (for cell viability assays). The cells were then incubated overnight at 37 °C with 5% CO_2_ to allow cell adhesion. After seeding, the maintenance media was replaced by serum-free medium and the cells were incubated for 24 h under normothermic (37 °C) or hyperthermic (40.5 °C) conditions. For each 96-well plate, a positive control (1% Triton X-100) was also considered.

### 4.3. Cell Viability Assays

The effect of temperature on metabolic activity of PMH was determined using the MTT reduction assay, as described in a previous work [32]. In order to evaluate the effect of temperature in the cell membrane disruption, the release of lactate dehydrogenase (LDH) to the extracellular medium was assessed using a protocol previously described by Valente et al. [33]. For both assays data were normalized to a no-effect (PMH at 37 °C) and a maximum-effect (PMH lysed with 1% Triton X-100) controls.

### 4.4. Collection, Preparation, and Analysis of Samples for Metabolomic Analysis

The collection of samples was performed according to a protocol used in a previous study [32]. Briefly, for the analysis of the extracellular volatile fraction, the culture medium from each well was collected on ice and subsequently centrifuged (2000× *g*, 5 min, 4 °C) to eliminate possible cellular fragments. Adherent cells were washed twice with 0.9% NaCl, and then an ice-cold methanol:water solution (80:20, *v/v*) was added to extract the intracellular metabolites. In sequence, cells were scraped, harvested, sonicated on ice for a few seconds, and centrifuged for 10 min at 3000× *g* at 4 °C. The supernatant was collected in a glass vial for further intracellular metabolome analysis. For each GC-MS procedure, quality control (QC) samples were prepared by pooling the same amount of each sample used in the study. All samples were kept at −80 °C until analysis.

The analysis of volatile fraction of the extracellular metabolome was performed by two methodologies based on headspace solid-phase microextraction (HS-SPME) coupled to GC-MS previously optimized by our group [34]. The analysis VOCs was carried out directly in the headspace of the cell culture medium, while VCCs were determined after a previous derivatization step. Sample preparation and GC-MS analysis of samples is described in detail in previous studies of our group [32,34].

### 4.5. GC-MS Data Pre-Processing

The GC-MS data were converted to the CDF file format using the software MASSTransit 3.0.1.16 (Palisade Corp, Newfield, NY) and pre-processed using the software MZmine 2.23 [35]. The parameters used in the pre-processing steps were set as follows: RT range 4.3–24.5 min, *m/z* range 50–400, MS data noise level 3 × 10^4^, m/z tolerance 0.5, baseline level 8 × 10^4^ and peak duration range 0.02–0.35 min for the intracellular analysis; RT range 2.1–25.0 min, *m*/*z* range 40–300, MS data noise level 1 × 10^5^, *m/z* tolerance 0.5, baseline level 4 × 10^4^ and peak duration range 0.02–0.3 for the VOCs analysis; and RT range 10.5–35.5 min, *m*/*z* range 50–500, MS data noise level 1 × 10^5^, *m*/*z* tolerance 0.5, baseline level 2 × 10^4^ and peak duration range 0.02–0.5 min for the VCCs analysis. After pre-processing steps, data were normalized by total chromatogram area to eliminate systematic and biological bias [36]. All known artefacts including peaks from the chromatographic column, SPME fibers (e.g., phthalates and siloxanes) and plasticizers, as well as chromatographic peaks with a signal to noise less than three and with relative standard deviation (RSD) higher than 30% across all QCs, were not considered in the statistical analysis.

### 4.6. Multivariate and Univariate Statistical Analysis

The final matrices were imported into the SIMCA-P 13.0.3 software (Umetrics Umea, Sweden) for multivariate analysis. Principal component analysis (PCA) and orthogonal projections to latent structures discriminant analysis (OPLS-DA) were applied to Pareto scaled data, with a default 7-fold internal cross validation, from which R^2^ and Q^2^ values reflect, respectively, the explained variance and the predictive capability of the models [37]. Simultaneously, all OPLS-DA models were validated through permutation test (500 permutations) and CV-ANOVA *p*-value (cross-validated analysis of variance) were also performed to determine the level of significance of group separation, a *p*-value < 0.05 being indicative of a significant model [37]. The variables (*m*/*z*-RT pairs) relevant for groups separation were assessed through the inspection of loading *S*-plots. Only the variables corresponding to the metabolite fingerprint (based on relative abundance and selectivity) and that simultaneously presented variables importance to the projection (VIP) > 1 and p(corr) >|0.5| were used in subsequent univariate analysis [37]. In addition, metabolites that resulted in multiple chromatographic peaks as a consequence of derivatization reactions were summed, as suggested by Mastrangelo et al. [38]. The statistical significance between the mean of two groups under study (PMH under normothermic vs. hyperthermic conditions) was assessed for the relevant metabolites (|p(corr)| >0.5 and VIP > 1) in GraphPad Prism version 6 (GraphPad Software, San Diego, CA, USA). The *p*-value was determined through an unpaired student *t*-test for normal distribution data or an unpaired Mann–Whitney test for a non-normal distribution. False discovery rate (FDR) corrected *p*-values were considered in the assessment of statistical significance [39]. Additionally, the effect size (ES), corrected for a small number of samples, were also determined for each relevant metabolite, according to equations provided in the literature [40].

### 4.7. Identification of Discriminant Metabolites

The identification of discriminant metabolites was done according to the Metabolomics Standards Initiative (MSI) guidelines, being based on the comparison of the retention index (RI) determined for each metabolite with the RI described in the literature and by comparing the retention time (RTs) and mass spectrum of the discriminant metabolite with spectra accessible in the National Institute of Standards and Technology (NIST14) mass spectral library [14]. Only for reverse match factors greater than 700, the tentative metabolite identification was considered. Whenever possible, the identification was unequivocally confirmed with authentic reference standards injected under the same chromatographic conditions. Metabolites that do not meet these identification criteria are reported throughout the paper according to their crescent RT values as ‘IM_i_’ (for the intracellular metabolites), ‘VOC_i_’ or ‘VCC_i_’ (i = 1, 2, 3…).

### 4.8. Biochemical Interpretation

Metabolic pathway analysis was used to identify biochemical pathways associated with alterations caused by the temperature increase. Metabolites significantly altered (*p* < 0.05) with Human Metabolome Database (HMDB) codes were imported into a Metaboanalyst 4.0 software (http://www.metaboanalyst.ca) and were searched against *Mus musculus* database [41]. Biochemical pathways were selected according to the *p*-value (*p* < 0.05) and pathway impact value (>0.1). The Human Metabolome Database (HMDB, www.hmdb.ca) and Kyoto Encyclopedia of Genes and Genomes (KEGG, www.kegg.jp) were also checked to support the biochemical interpretation. Furthermore, to search for possible correlations between metabolites significantly altered (*p* < 0.01), Spearman’s rank correlation coefficient was also calculated and represented in a heatmap.

## 5. Conclusions

Heat stress is a life-threatening condition capable of disturbing cellular homeostasis. In this work, we presented a metabolomic study of the liver following hyperthermia in an in vitro model. Our data revealed that GC-MS metabolomic profiling can be successfully used to visualize the hyperthermia-induced disorders, since in the present study prominent derangements were observed in the intra and extracellular hepatic metabolome. Multivariate and univariate statistical analysis revealed a high number of compromised metabolites that are essentially associated with the energetic pathway, synthesis of antioxidant defenses, and with the lipid peroxidation process. Taking into account the results obtained, it is our belief that this metabolomic study may represent an interesting platform to evaluate and understand the deleterious effects of heat stroke in humans.

## Supplementary Materials

The following are available online at https://www.mdpi.com/2218-1989/9/10/228/s1, Figure S1: Principal component analysis (PCA) score scatter plots obtained for the GC-MS chromatograms of the three distinct procedures ((A) intracellular metabolite profiling, (B) extracellular metabolite profiling—VOCs and (C) extracellular metabolite profiling—VCCs) to evaluate data quality. Each sample is represented in the score’s scatter plot as an individual variable, namely quality control (QC) samples (●) and the intracellular/extracellular content of all primary mouse hepatocytes samples (●). (D) Boxplot of the three internal standards used in the different metabolomics studies. Data are expressed as the mean and standard deviation (SD) of the normalized peak area by total area of the chromatogram. All internal standards presented a variation coefficient inferior to 20%.; Figure S2: PCA score scatter plots obtained for the chromatograms corresponding to cells exposed to normothermic (*n* = 10, ●) and hyperthermic (*n* = 10, ▲) conditions, after analysis of the (A) intracellular metabolome, as well as (B) VOCs and (C) VCCs present in the extracellular metabolome.; Table S1. Identification of discriminant intracellular metabolites selected from OPLS-DA loading *S*-plots (VIP > 1 and |p(corr)| >0.5). The identification of the metabolites was done according to the Metabolomics Standards Initiative (MSI) levels. They were characterized by retention time (RT), characteristic ions (*m/z*), retention index (from the literature (*RI*_lit_) and compared with the calculated (*RI*_calc_) for the same chromatographic column), reverse match factor from National Institute of Standards and Technology (NIST) and Human Metabolome Database (HMDB) and Kyoto Encyclopedia of Genes and Genomes (KEGG) code (when available). Table S2. Identification of discriminant volatile extracellular metabolites (VOCs and VCCs) selected from OPLS-DA loading *S*-plots (VIP > 1 and |p(corr)| >0.5). The identification of the metabolites was done according to the MSI levels. They were characterized by retention time (RT), characteristic ions (*m/z*), retention index (from the literature (*RI*_lit_) and compared with the calculated (*RI*_calc_) for the same chromatographic column), reverse match factor from NIST and HMDB and KEGG code (when available).
