# Supplementary material for: Hepatic Metabolic Derangements Triggered by Hyperthermia: An In Vitro Metabolomic Study
Source: Metabolites. 2019 Oct 15;9(10):228. doi: 10.3390/metabo9100228 (PMC6835778; doi:10.3390/metabo9100228)
Supplement: Supplementary file 1 [file metabolites-09-00228-s001.pdf]

Hepatic metabolic derangements triggered by hyperthermia: an *in vitro* metabolomic study. *Metabolites*. Araújo, A.M.; Enea, M.; Carvalho, F.; Bastos, M.L.; Carvalho, M. and Guedes de Pinho, P.. Corresponding author: Ana Margarida Araújo (ana.margarida.c.araujo@gmail.com). UCIBIO/REQUIMTE, Laboratory of Toxicology, Faculty of Pharmacy, University of Porto, Rua Jorge Viterbo Ferreira, 228, 4050-313, Porto, Portugal

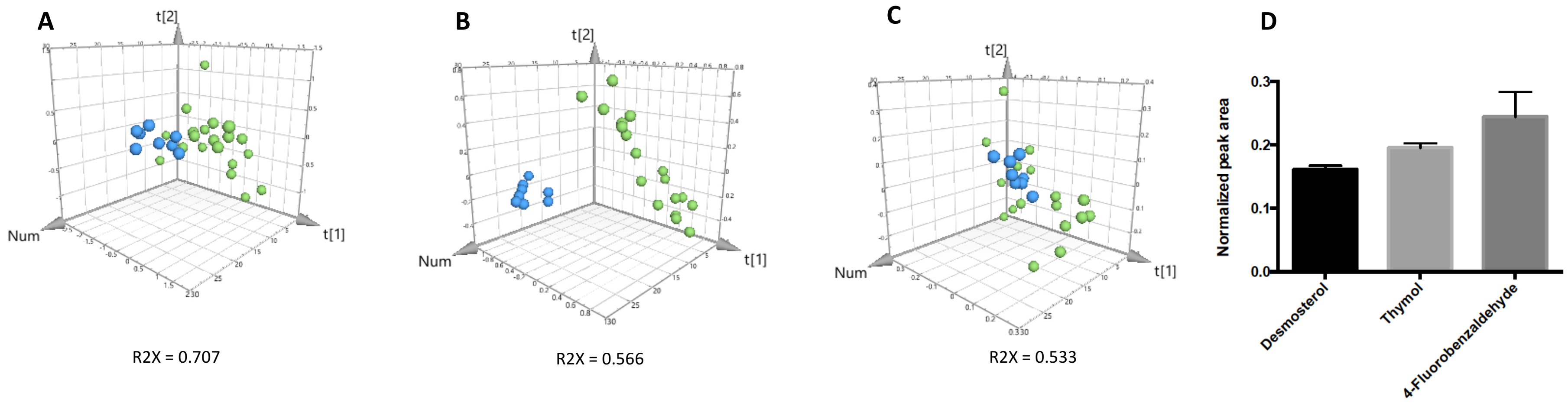

Figure S1. PCA scores scatter plots obtained for the GC-MS chromatograms of the three distinct procedures ((A) intracellular metabolite profiling, (B) extracellular metabolite profiling – VOCs and (C) extracellular metabolite profiling – VCCs) to evaluate data quality. Each sample is represented in the scores scatter plot as an individual variable, namely QCs samples (●) and the intracellular/extracellular content of all primary mouse hepatocytes samples (●). (D) Boxplot of the three internal standards used in the different metabolomics studies. Data are expressed as the mean and standard deviation (SD) of the normalized peak area by total area of the chromatogram. All internal standards presented a variation coefficient inferior to 20%.

Hepatic metabolic derangements triggered by hyperthermia: an *in vitro* metabolomic study. *Metabolites*. Araújo, A.M.; Enea, M.; Carvalho, F.; Bastos, M.L.; Carvalho, M. and Guedes de Pinho, P.. Corresponding author: Ana Margarida Araújo (ana.margarida.c.araujo@gmail.com). UCIBIO/REQUIMTE, Laboratory of Toxicology, Faculty of Pharmacy, University of Porto, Rua Jorge Viterbo Ferreira, 228, 4050-313, Porto, Portugal

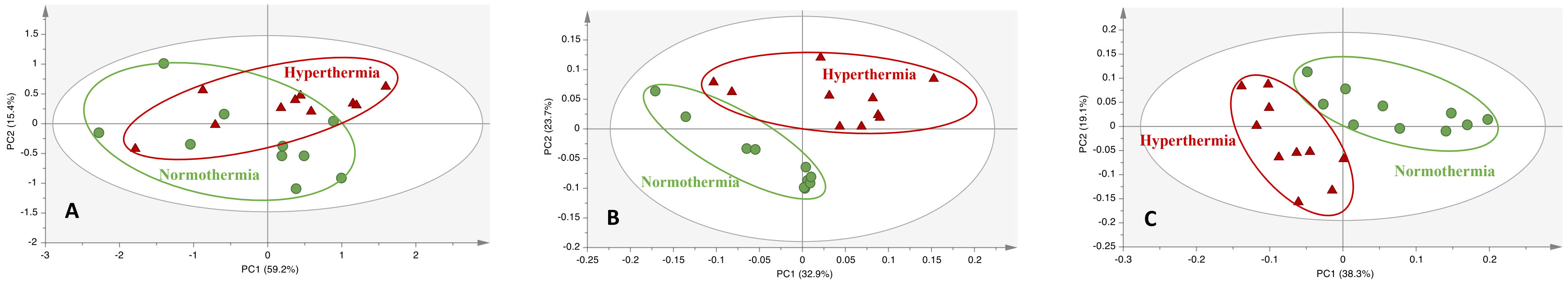

Figure S2. PCA scores scatter plots obtained for the chromatograms corresponding to cells exposed to normothermic (*n*=10, ●) and hyperthermic (*n*=10, ▲) conditions, after analysis of the (A) intracellular metabolome, as well as (B) VOCs and (C) VCCs present in the extracellular metabolome.

Table S1. Identification of discriminant intracellular metabolites selected from OPLS-DA loading *S*-plots (VIP>1 and |p(corr)|>0.5). The identification of the metabolites was done according to the MSI levels. They were characterized by retention time (RT), characteristic ions (m/z), retention index (from the literature (RI_{lit}) and compared with the calculated (RI_{calc}) for the same chromatographic column), reverse match factor from NIST and HMDB and KEGG code (when available).

Metabolite	RT (min)	Characteristic ions (m/z)	RI_{calc}	RI_{lit}	R. Match	HMDB ID	KEGG ID	ID level
L-Lactate, 2TMS	5.615	73+117+147	1055	1061	942	HMDB00190	C00186	L1
L-Isoleucine, TMS	6.996	69+73+86	1179	1179	785	HMDB00172	C00407	L1
L-Valine, 2TMS	7.342	73+144+147	1211	1224	913	HMDB00883	C00183	L1
L-Isoleucine, 2TMS	8.106	73+158+159	1287	1272	929	HMDB00172	C00407	L1
Fumarate, 2TMS	8.646	73+147+245	1344	1348	945	HMDB00134	C00122	L1
L-Aspartate, 2TMS	9.348	73+75+160	1421	1420	751	HMDB00191	C00049	L1
Aminomalonic acid, 3TMS	9.711	73+147+218	1463	1485	834	HMDB01147	C00872	L1
Malate, 2TMS	9.834	73+147+233	1478	1477	938	HMDB00744	C00711	L1
L-Aspartate, 3TMS	10.112	73+100+232	1510	1512	932	HMDB00191	C00049	L1
2-Ketoglutarate, 2TMS	10.559	73+75+147	1566	1587	825	HMDB02812	C00026	L1
L-Glutamate, 3TMS	10.921	73+128+246	1611	1632	916	HMDB00148	C00025	L1
L-Phenylalanine, 2TMS	11.022	73+192+218	1624	1625	904	HMDB00159	C00079	L1

D-Arabinose, 4TMS	11.338	73+103+217	1664	1654	845	HMDB29942	_	L2
L-Ornithine, 4TMS	12.410	73+142+174	1811	1798	880	HMDB00214	C00077	L1
1,5-Anhydrohexitol, 4TMS	12.480	73+147+217	1821	1843	736	_	_	L2
D-Glucuronic acid γ-lactone, 3TMS	13.204	73+147+160	1927	1921	726	HMDB06355	C02670	L2
Mannitol, 6TMS	13.397	73+147+319	1956	1958	751	HMDB01363	C00392	L2
D-Gluconic acid, 6TMS	13.721	73+147+217	2005	2039	870	HMDB00625	C00257	L1
Myo-inositol, 6TMS	14.238	73+147+217	2084	2113	906	HMDB00211	C00137	L1
D-Ribose, 5TMS	14.284	73+217+315	2092	_	773	HMDB00283	C00121	L2
2-Palmitoylglycerol, 2TMS	18.280	73+129+147	2549	2558	803	_	_	L2
Docosahexaenoic acid, TMS	18.350	73+79+91	2557	2562	811	HMDB02183	C06429	L2
2-Monostearin, 2TMS	19.892	73+129+147	2740	2772	814	_	_	L2
Glycerol monostearate, 2TMS	20.178	73+147+399	2778	2806	903	_	_	L2
IM 1	5.384	73+152+318	1036	_	_	_	_	L4
IM 2	5.492	73+74+174	1045	_	_	_	_	L4
IM 3	11.400	73+75+115	1672	_	_	_	_	L4
IM 4 – carbohydrate derivative	12.549	50+76+84	1831	_	_	_	_	L3
IM 5 – carbohydrate derivative	13.444	73+149+217	1963	_	_	_	_	L3

IM 6 – carbohydrate derivative	16.359	73+75+147	2341	_	_	_	_	L3

Notes:

IM – Intracellular metabolite

L1 (Level 1) – Metabolites unequivocally identified by standards

L2 (Level 2) – Putatively annotated compounds (i.e. identification was based only in the mass spectrum similarity with commercial spectral libraries, retention indexes and reverse percentage of match)

L3 (Level 3) – Putatively characterized compound classes (i.e. spectral and/or physicochemical properties are consistent with a particular class of compounds)

L4 (Level 4) – Unknown compounds (although unidentified or unclassified metabolites can still be differentiated based upon mass spectrum data)

Table S2. Identification of discriminant volatile extracellular metabolites (VOCs and VCCs) selected from OPLS-DA loading *S*-plots (VIP>1 and |p(corr)|>0.5). The identification of the metabolites was done according to the MSI levels. They were characterized by retention time (RT), characteristic ions (m/z), retention index (from the literature (RI_{lit}) and compared with the calculated (RI_{calc}) for the same chromatographic column), reverse match factor from NIST and HMDB and KEGG code (when available).

Metabolite	RT (min)	Characteristic ions (m/z)	RI_{calc}	RI_{lit}	R. Match	HMDB ID	KEGG ID	ID level
Extracellular metabolites – VOCs								
2-Pentanone	3.112	43+58+71	_	673	873	HMDB34235	C01949	L1
4-Methyl-2-pentanone	3.895	41+43+57	_	716	889	_	_	L1
2-Hexanone	4.844	41+43+57	_	771	864	_	_	L1
Hexanal	5.103	41+43+44	803	806	890	HMDB05994	C02373	L1
1,1-Dimethylpropyl acetate	5.278	43+55+70	810	800	800	_	_	L2
2,4-Dimethyl-1-heptene	6.079	41+43+70	840	836	910	_	_	L2
1-Hexanol	6.835	41+43+56	869	860	868	HMDB12971	C00854	L1
Cyclohexanol	7.314	44+57+67	887	856	853	_	_	L2
Heptanal	7.721	41+43+44	902	905	845	HMDB31475	C14390	L1
3-Octenol	10.036	43+57+72	980	980	760	_	_	L1
Octanal	10.714	41+43+56	1002	1005	859	HMDB01140	C01545	L1

3-Ethyl-4-methylpentanol	11.250	41+55+69	1020	1020	955	HMDB0059859	_	L2
2-Ethyl-1-hexanol	11.496	41+43+57	1028	1010	915	HMDB0031231	C02498	L2
2-Nonanone	12.214	41+43+58	1052	1052	900	HMDB0013822	_	L1
Acetophenone	12.595	51+77+105	1064	1052	947	HMDB0033910	C07113	L1
1-Octanol	12.770	41+43+56	1070	1059	879	HMDB0001183	C00756	L1
4-Methylbenzaldehyde	13.138	65+91+119	1082	1095	924	HMDB0029638	C06758	L1
1-(2,4-dimethyl-3-furyl)ethanone	13.306	43+67+123	1087	1057	843	_	_	L2
2,4,6-Trimethyldecane	13.461	43+57+71	1092	1121	817	_	_	L2
Nonanal	13.785	41+43+56	1103	1104	921	HMDB0059835	_	L1
Isoborneol	15.801	41+43+95	1172	1174	758	HMDB0035819	_	L2
Decanal	16.765	41+43+55	1205	1204	888	HMDB0011623	C12307	L1
2,5-Dimethylbenzaldehyde	17.044	91+105+133	1215	1208	916	_	_	L2
1-(2,4,6-Trimethylphenyl)ethanone	19.334	91+119+147	1296	1312	858	_	_	L2
VOC 1	5.633	43+45+49	823	_	_	_	_	L4
VOC 2	8.128	41+42+59	916	_	_	_	_	L4
VOC 3	11.335	43+55+83	1023	_	_	_	_	L4
VOC 4	11.832	43+109+110	1039	_	_	_	_	L4

VOC 5	12.964	55+57+69	1076	_	_	_	_	L4
VOC 6	14.567	43+121+123	1130	_	_	_	_	L4
VOC 7	17.762	43+57+71	1240	_	_	_	_	L4
Extracellular metabolites – VCCs								
Formaldehyde	10.783	195	1008	_	909	HMDB0001426	C00067	L1
Acetone	15.534	236+253	1164	1167	916	HMDB0001659	C00207	L1
Propanal	16.063/16.257	236	1182/1189	1193	916/916	HMDB0003366	C00479	L1
2-Butanone	17.561/17.658	250+267	1234/1238	_	850/852	HMDB0000474	C02845	L1
Butanal	18.504/18.686	239	1268/1274	1287	873/842	HMDB0003543	C01412	L1
2,7,10-Trimethyldodecane	18.737	43+57+71	1276	_	858	HMDB0062790	_	L2
2-Pentanone	19.524/19.727	253+281	1304/1312	_	849/869	HMDB0034235	C01949	L1
2-Methylbutanal	19.605	239	1307	_	809	HMDB0031526	C02223	L1
3-Methylbutanal	19.884/20.091	239+266+281	1318/1326	1328	853/805	HMDB0006478	C07329	L1
Tridecane	20.011	43+57+71	1323	1300	879	HMDB0034284	C13834	L2
Pentanal	20.954/21.115	239	1358/1364	1369	884/880	HMDB0031206	_	L1
2-Hexanone	21.678/21.941	253	1385/1395	_	840/847	HMDB0005842	_	L1
Tetradecane	22.101	43+57+71	1401	1400	911	HMDB0059907	_	L2

3-Methyl-2-butenal	23.037/23.304	264+279	1438/1449	1442	850/812	HMDB0012157	C07330	L2
Hexanal	23.359/23.494	239	1451/1457	1460	856/790	HMDB0005994	C02373	L1
1-Dodecanol	23.943	43+55+69	1474	1473	905	HMDB0011626	C02277	L2
2-Heptanone	24.201/24.862	253	1485/1512	_	800/774	HMDB0003671	C08380	L1
5-Hexen-2-one	25.179	55+82	1525	1523	730	_	_	L2
Heptanal	25.667/25.751	239	1545/1549	1558	814/813	HMDB0031475	C14390	L1
Octanal	27.898/27.961	239	1641/1644	1654	840/778	HMDB0001140	C01545	L1
Benzaldehyde	28.371/28.549	301	1662/1670	1672	843/858	HMDB0006115	C00193	L1
Glyoxal	33.869/33.958/34.038	235+448	1923/1928/1932	1935	928/913/921	_	_	L1
Methylglyoxal	34.136/34.326/34.639	265	1937/1947/1963	_	931/885/935	HMDB0001167	C00546	L1
VCC 1	19.380	161+117+195	1299	_	_	_	_	L4
VCC 2	19.440	43+55+69	1301	_	_	_	_	L4
VCC 3	20.320	161+181	1334	_	_	_	_	L4
VCC 4	20.493	43+57+71	1341	_	_	_	_	L4
VCC 5	20.908	71+161+181	1356	_	_	_	_	L4
VCC 6	23.206	181+194	1445	_	_	_	_	L4
VCC 7	25.002	43+69	1517	_	_	_	_	L4

VCC 8	25.429	43+57+71	1535	_	_	_	_	L4
VCC 9	25.954	266+282	1557	_	_	_	_	L4
VCC 10	27.512	181+233	1624	_	_	_	_	L4
VCC 11 – ketone derivative	30.205	181+253	1745	_	_	_	_	L3
VCC 12	30.773	285+315	1772	_	_	_	_	L4
VCC 13	35.101	236+279	1986	_	_	_	_	L4

Notes:

L1 (Level 1) – Metabolites unequivocally identified by standards

L2 (Level 2) – Putatively annotated compounds (i.e. identification was based only in the mass spectrum similarity with commercial spectral libraries, retention indexes and reverse percentage of match)

L3 (Level 3) – Putatively characterized compound classes (i.e. spectral and/or physicochemical properties are consistent with a particular class of compounds)

L4 (Level 4) – Unknown compounds (although unidentified or unclassified metabolites can still be differentiated based upon mass spectrum data)
